# 2-Chloro-*N*-ethyl-9-isopropyl-9*H*-purin-6-amine

**DOI:** 10.1107/S1600536812035933

**Published:** 2012-08-23

**Authors:** Federico Andrés Giovagnoli, Michal Rouchal, Peter Bartoš, Robert Vícha

**Affiliations:** a Department of Chemistry, Faculty of Technology, Tomas Bata University in Zlin, Nám. T. G. Masaryka 275, Zlín, 762 72, Czech Republic; b Department of Chemistry, Faculty of Science, Masaryk University, Kamenice 5, Brno-Bohunice, 625 00, Czech Republic

## Abstract

In the title compound, C_10_H_14_ClN_5_, the purine ring system is essentially planar, with an r.m.s. deviation from the least-squares plane defined by the nine constituent atoms of 0.0063 (11) Å. In the crystal, mol­ecules are linked by weak N—H⋯N and C—H⋯π inter­actions.

## Related literature

For the synthesis, see: Fiorini & Abel (1998 ▶). For related structures, see: Kubicki & Codding (2001 ▶); Rouchal *et al.* (2009*a* ▶,*b* ▶, 2010 ▶). For other related literature, see: Legraverend & Grierson (2006 ▶).
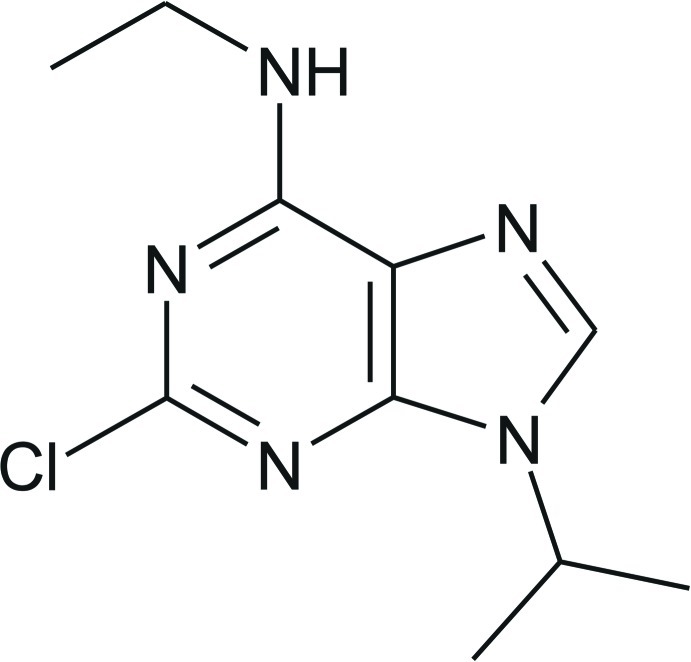


## Experimental

### 

#### Crystal data

C_10_H_14_ClN_5_
*M* *_r_* = 239.71
Monoclinic, 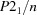

*a* = 8.1385 (2) Å
*b* = 9.6245 (2) Å
*c* = 14.8388 (3) Å
β = 92.997 (2)°
*V* = 1160.72 (4) Å^3^
*Z* = 4
Mo *K*α radiation
μ = 0.31 mm^−1^
*T* = 120 K
0.40 × 0.40 × 0.20 mm

#### Data collection

Oxford Diffraction Xcalibur diffractometer with a Sapphire2 (large Be window) detector
Absorption correction: multi-scan *CrysAlis RED* (Oxford Diffraction, 2009 ▶) *T*_min_ = 0.973, *T*_max_ = 1.000
13543 measured reflections
2041 independent reflections
1793 reflections with *I* > 2σ(*I*)
*R*_int_ = 0.015

#### Refinement

*R*[*F* ^2^ > 2σ(*F* ^2^)] = 0.025
*wR*(*F* ^2^) = 0.069
*S* = 1.07
2041 reflections
152 parameters
H atoms treated by a mixture of independent and constrained refinement
Δρ_max_ = 0.21 e Å^−3^
Δρ_min_ = −0.22 e Å^−3^

### 

Data collection: *CrysAlis CCD* (Oxford Diffraction, 2009 ▶); cell refinement: *CrysAlis RED* (Oxford Diffraction, 2009 ▶); data reduction: *CrysAlis RED*; program(s) used to solve structure: *SHELXS97* (Sheldrick, 2008 ▶); program(s) used to refine structure: *SHELXL97* (Sheldrick, 2008 ▶); molecular graphics: *ORTEP-3* (Farrugia, 1997 ▶) and *Mercury* (Macrae *et al.*, 2008 ▶); software used to prepare material for publication: *SHELXL97*.

## Supplementary Material

Crystal structure: contains datablock(s) global, I. DOI: 10.1107/S1600536812035933/lx2254sup1.cif

Structure factors: contains datablock(s) I. DOI: 10.1107/S1600536812035933/lx2254Isup2.hkl

Supplementary material file. DOI: 10.1107/S1600536812035933/lx2254Isup3.cml

Additional supplementary materials:  crystallographic information; 3D view; checkCIF report

## Figures and Tables

**Table 1:** Hydrogen-bond geometry (Å, °) *Cg*1 is the center of gravity of the pyridine ring (C1/N1/C2–C4/ N2).

*D*—H⋯*A*	*D*—H	H⋯*A*	*D*⋯*A*	*D*—H⋯*A*
N3—H3⋯N4^i^	0.837 (16)	2.205 (17)	2.9979 (16)	158.2 (15)
C8—H8⋯*Cg*1^ii^	1.00 (1)	2.90 (1)	3.6403 (13)	131 (1)

Symmetry codes: (i) 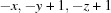; (ii) 
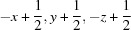
.

**Fig. 1.**
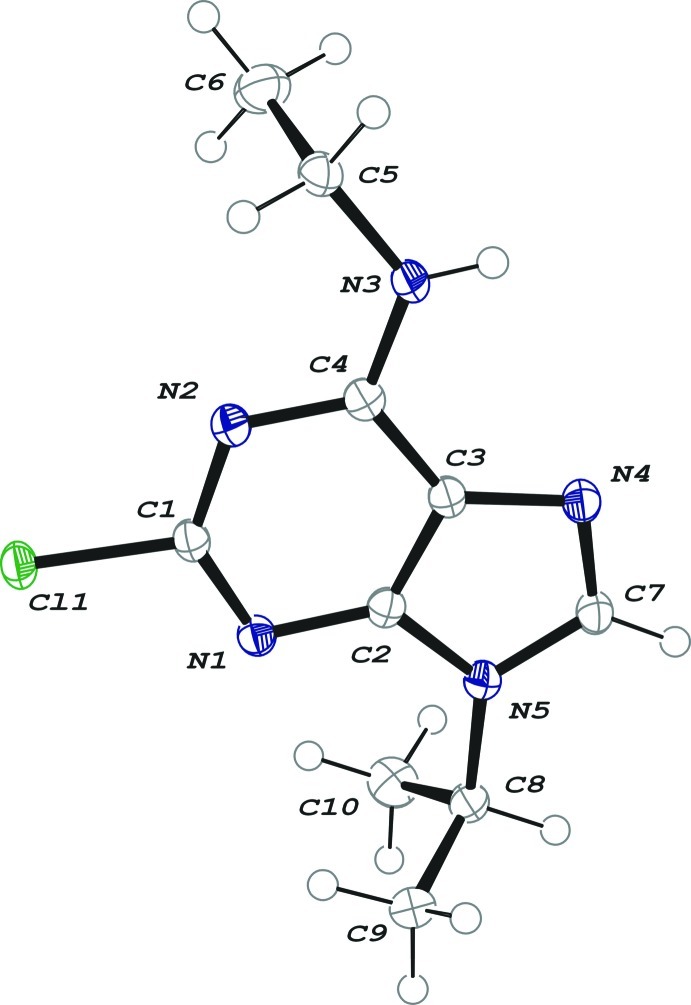
The molecular structure of the title compound with the atom numbering scheme. Displacement ellipsoids are drawn at the 50% probability level. H atoms are presented as small spheres of arbitrary radius.

**Fig. 2.**
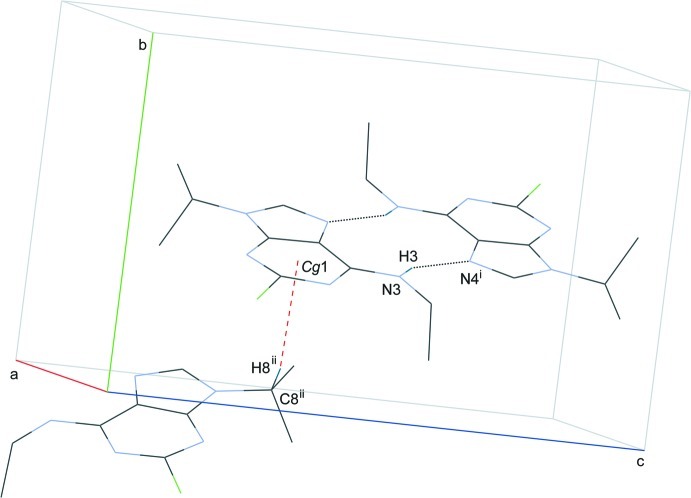
A view of the N—H···N (black dotted lines) and C—H···π (red dashed lines) interactions in the crystal structure of the title compound. H atoms non-participating in hydrogen-bonding were omitted for clarity. Symmetry codes: (i) -x, -y + 1, -z + 1; (ii) -x + 0.5, y - 0.5, -z + 0.5.

## supplementary crystallographic information

### Comment

The wide range of biological activities of di-, tri-, or tetrasubstituted purines is closely associated with essentially unlimited number of substituents that can be combined in the C2, C6, C8 and N9 positions of the purine ring (Legraverend & Grierson, 2006). The title molecule has been prepared as a part of our ongoing study of novel 2,6,9-trisubstituted purine series (Rouchal *et al.*, 2009*a*,*b*, 2010). To the best of our knowledge, the title compound has not been described in the literature so far.

In the title molecule (Fig. 1), the purine unit is essentially planar, with a r.m.s. deviation of 0.0063 (11) Å from the least-squares plane defined by the nine constituent atoms. Although all pyrimidine atoms lie essentially in plane, the ring is markedly deformed from regular hexagon geometry with N1–C2–N3 angle of 132.0 (11)°. In the crystal structure (Fig. 2), molecules are connected by weak intermolecular N—H···N and C—H···π interactions (Table 1).

### Experimental

The title compound was prepared according to slightly modified literature procedure (Fiorini & Abel, 1998). 2,6-Dichloro-9-isopropyl-9*H*-purine (100 mg, 0.43 mmol) and ethylamine hydrochloride (37 mg, 0.45 mmol) were dissolved in the mixture of DMF (3 cm^3^) and triethylamine (87 mg, 0.86 mmol). The resulting solution was stirred at 90 °C for 3 h. Subsequently, the mixture was diluted with water and extracted with ethyl acetate (6 × 10 cm^3^). Combined organic layers were washed twice with brine, dried over Na_2_SO_4_ and evaporated in vacuum. Crystallization of the crude product from diethyl ether at room temperature provided desired compound as colorless crystals (67 mg, 65%, mp 390–393 K) suitable for X-ray diffraction analysis.

### Refinement

All carbon bound H atoms were placed at calculated positions and were refined as riding with their *U*_iso_ set to either 1.2*U*_eq_ or 1.5*U*_eq_ (methyl) of the respective carrier atoms. The positions of methyl H atoms were optimized rotationally. Nitrogen bound H atom was located in a difference Fourier map and refined freely.

### Crystal data

**Table:**

C_10_H_14_ClN_5_	*F*(000) = 504
*M**_r_* = 239.71	*D*_x_ = 1.372 Mg m^−^^3^
Monoclinic, *P*2_1_/*n*	Melting point: 392 K
Hall symbol: -P 2yn	Mo *K*α radiation, λ = 0.71073 Å
*a* = 8.1385 (2) Å	Cell parameters from 9070 reflections
*b* = 9.6245 (2) Å	θ = 2.9–27.2°
*c* = 14.8388 (3) Å	µ = 0.31 mm^−^^1^
β = 92.997 (2)°	*T* = 120 K
*V* = 1160.72 (4) Å^3^	Block, colourless
*Z* = 4	0.40 × 0.40 × 0.20 mm

### Data collection

**Table:**

Oxford Diffraction Xcalibur diffractometer with a Sapphire2 (large Be window) detector	2041 independent reflections
Radiation source: Enhance (Mo) X-ray Source	1793 reflections with *I* > 2σ(*I*)
Graphite monochromator	*R*_int_ = 0.015
Detector resolution: 8.4353 pixels mm^-1^	θ_max_ = 25.0°, θ_min_ = 2.9°
ω scan	*h* = −9→9
Absorption correction: multi-scan *CrysAlis RED* (Oxford Diffraction, 2009)	*k* = −10→11
*T*_min_ = 0.973, *T*_max_ = 1.000	*l* = −17→17
13543 measured reflections	

### Refinement

**Table:**

Refinement on *F*^2^	0 restraints
Least-squares matrix: full	H atoms treated by a mixture of independent and constrained refinement
*R*[*F*^2^ > 2σ(*F*^2^)] = 0.025	*w* = 1/[σ^2^(*F*_o_^2^) + (0.0372*P*)^2^ + 0.3781*P*] where *P* = (*F*_o_^2^ + 2*F*_c_^2^)/3
*wR*(*F*^2^) = 0.069	(Δ/σ)_max_ = 0.001
*S* = 1.07	Δρ_max_ = 0.21 e Å^−^^3^
2041 reflections	Δρ_min_ = −0.22 e Å^−^^3^
152 parameters	

### Special details

**Table:**

Experimental. Spectral properties of title compound:1H NMR (CDCl_3_): 1.30 (t, J = 7.3 Hz, 3H, NHCH_2_CH_3_), 1.57 (d, J = 6.6 Hz, 6H, CH(CH_3_)_2_), 3.72 (m, 2H, NHCH_2_CH_3_), 4.80 (septet, J = 6.9 Hz, 1H, CH(CH_3_)_2_), 5.84 (bs, 1H, NHCH_2_CH_3_), 7.76 (s, 1H, NC8HN) p.p.m.. 13 C NMR (CDCl_3_): 15.1(CH_3_), 23.0(CH_3_), 36.0(CH_2_), 47.1(CH), 119.0(C), 137.6(CH), 154.5(C), 155.5(C) p.p.m.. IR (KBr): 3103(*m*), 2974(*m*), 2933(w), 1726(w), 1605(*s*), 1560(*m*), 1481(w), 1454(w), 1435(w), 1404(*m*), 1362(*m*), 1309(*s*), 1227(*s*), 1188(w), 130(w), 1034(*s*), 941(*m*), 874(*m*), 785(*m*), 657(*m*), 609(w) cm^-1^. GC—EI—MS (200 °C, 70 eV): 241(*M*^+^(^37^Cl), 30), 240 (13), 239(*M*^+^(^35^Cl), 95), 226 (20), 225 (7), 224 (62), 213 (10), 211 (32), 199 (9), 198 (18), 197 (27), 196 (45), 184 (32), 183 (9), 182 (100), 171 (17), 169 (54), 161 (27), 160 (17), 156 (6), 155 (10), 154 (19), 153 (8), 146 (20), 134 (43), 133 (22), 120 (6), 119 (47), 118 (5), 108 (9), 107 (12), 106 (7), 93 (8), 92 (21), 80 (7), 68 (5), 67 (9), 66 (11), 65 (6), 55 (6), 54 (12), 53 (9), 44 (74), 43 (34), 42 (11), 41 (34), 40 (6) m/*z*(%). Elemental analysis calc for C_10_H_14_ClN_4_ (239.70) C 50.11, H 5.89, N 29.22; found C 50.16, H 5.92, N 29.02.
Geometry. All e.s.d.'s (except the e.s.d. in the dihedral angle between two l.s. planes) are estimated using the full covariance matrix. The cell e.s.d.'s are taken into account individually in the estimation of e.s.d.'s in distances, angles and torsion angles; correlations between e.s.d.'s in cell parameters are only used when they are defined by crystal symmetry. An approximate (isotropic) treatment of cell e.s.d.'s is used for estimating e.s.d.'s involving l.s. planes.
Refinement. Refinement of *F*^2^ against ALL reflections. The weighted *R*-factor *wR* and goodness of fit *S* are based on *F*^2^, conventional *R*-factors *R* are based on *F*, with *F* set to zero for negative *F*^2^. The threshold expression of *F*^2^ > 2σ(*F*^2^) is used only for calculating *R*-factors(gt) *etc*. and is not relevant to the choice of reflections for refinement. *R*-factors based on *F*^2^ are statistically about twice as large as those based on *F*, and *R*- factors based on ALL data will be even larger.

### Fractional atomic coordinates and isotropic or equivalent isotropic displacement parameters (Å^2^)

**Table:**

	*x*	*y*	*z*	*U*_iso_*/*U*_eq_	
Cl1	0.71286 (4)	0.25662 (4)	0.37828 (2)	0.02326 (12)	
N1	0.46503 (13)	0.39081 (11)	0.30528 (7)	0.0176 (2)	
N2	0.45822 (13)	0.33371 (11)	0.46370 (7)	0.0172 (2)	
N3	0.24124 (14)	0.38987 (12)	0.55160 (7)	0.0195 (3)	
H3	0.147 (2)	0.4230 (17)	0.5565 (11)	0.027 (4)*	
N4	0.08494 (13)	0.52739 (11)	0.38017 (7)	0.0194 (3)	
N5	0.21933 (13)	0.51796 (11)	0.25145 (7)	0.0180 (2)	
C1	0.52134 (15)	0.33857 (13)	0.38306 (8)	0.0169 (3)	
C2	0.31722 (15)	0.45181 (13)	0.31630 (8)	0.0164 (3)	
C3	0.23370 (15)	0.45883 (13)	0.39493 (9)	0.0164 (3)	
C4	0.30943 (15)	0.39438 (13)	0.47204 (8)	0.0166 (3)	
C5	0.31812 (16)	0.31996 (15)	0.63006 (8)	0.0211 (3)	
H5A	0.2794	0.3635	0.6855	0.025*	
H5B	0.4388	0.3329	0.6301	0.025*	
C6	0.27990 (19)	0.16560 (16)	0.63171 (10)	0.0312 (4)	
H6A	0.3327	0.1238	0.6861	0.047*	
H6B	0.3218	0.1212	0.5781	0.047*	
H6C	0.1606	0.1521	0.6321	0.047*	
C7	0.08323 (16)	0.56029 (14)	0.29428 (9)	0.0199 (3)	
H7	−0.0051	0.6094	0.2644	0.024*	
C8	0.25198 (17)	0.53989 (14)	0.15553 (9)	0.0210 (3)	
H8	0.1594	0.5962	0.1277	0.025*	
C9	0.40995 (17)	0.62252 (15)	0.14745 (9)	0.0250 (3)	
H9A	0.4035	0.7089	0.1820	0.038*	
H9B	0.4244	0.6445	0.0839	0.038*	
H9C	0.5037	0.5674	0.1712	0.038*	
C10	0.25535 (19)	0.40217 (16)	0.10584 (10)	0.0285 (3)	
H10A	0.1506	0.3537	0.1119	0.043*	
H10B	0.3455	0.3448	0.1317	0.043*	
H10C	0.2722	0.4190	0.0418	0.043*	

### Atomic displacement parameters (Å^2^)

**Table:**

	*U*^11^	*U*^22^	*U*^33^	*U*^12^	*U*^13^	*U*^23^
Cl1	0.01873 (19)	0.0305 (2)	0.02086 (19)	0.00947 (13)	0.00355 (13)	0.00232 (13)
N1	0.0166 (6)	0.0174 (6)	0.0189 (6)	0.0010 (4)	0.0019 (4)	−0.0002 (4)
N2	0.0164 (5)	0.0172 (6)	0.0181 (5)	0.0006 (4)	0.0015 (4)	−0.0013 (4)
N3	0.0169 (6)	0.0237 (6)	0.0181 (6)	0.0048 (5)	0.0037 (4)	0.0010 (4)
N4	0.0170 (6)	0.0193 (6)	0.0220 (6)	0.0021 (4)	0.0018 (4)	0.0002 (5)
N5	0.0164 (6)	0.0199 (6)	0.0179 (5)	0.0021 (4)	0.0012 (4)	0.0019 (5)
C1	0.0148 (6)	0.0156 (7)	0.0203 (6)	0.0007 (5)	0.0019 (5)	−0.0015 (5)
C2	0.0158 (6)	0.0141 (7)	0.0191 (6)	−0.0012 (5)	−0.0004 (5)	−0.0003 (5)
C3	0.0151 (6)	0.0143 (7)	0.0200 (6)	−0.0007 (5)	0.0014 (5)	−0.0015 (5)
C4	0.0169 (6)	0.0136 (6)	0.0192 (6)	−0.0023 (5)	0.0010 (5)	−0.0027 (5)
C5	0.0202 (7)	0.0265 (8)	0.0166 (6)	0.0029 (6)	0.0020 (5)	0.0003 (5)
C6	0.0311 (8)	0.0295 (9)	0.0329 (8)	−0.0026 (6)	0.0016 (6)	0.0085 (6)
C7	0.0159 (6)	0.0200 (7)	0.0238 (7)	0.0025 (5)	0.0014 (5)	0.0015 (6)
C8	0.0202 (7)	0.0261 (8)	0.0169 (6)	0.0038 (5)	0.0008 (5)	0.0044 (5)
C9	0.0255 (7)	0.0241 (8)	0.0258 (7)	0.0015 (6)	0.0046 (6)	0.0065 (6)
C10	0.0300 (8)	0.0333 (9)	0.0220 (7)	−0.0012 (6)	−0.0001 (6)	−0.0046 (6)

### Geometric parameters (Å, º)

**Table:**

Cl1—C1	1.7516 (13)	C5—H5A	0.9900
N1—C1	1.3189 (16)	C5—H5B	0.9900
N1—C2	1.3561 (16)	C6—H6A	0.9800
N2—C1	1.3274 (16)	C6—H6B	0.9800
N2—C4	1.3558 (16)	C6—H6C	0.9800
N3—C4	1.3312 (17)	C7—H7	0.9500
N3—C5	1.4572 (17)	C8—C10	1.518 (2)
N3—H3	0.837 (17)	C8—C9	1.5217 (19)
N4—C7	1.3126 (17)	C8—H8	1.0000
N4—C3	1.3861 (17)	C9—H9A	0.9800
N5—C7	1.3677 (17)	C9—H9B	0.9800
N5—C2	1.3733 (16)	C9—H9C	0.9800
N5—C8	1.4766 (16)	C10—H10A	0.9800
C2—C3	1.3824 (18)	C10—H10B	0.9800
C3—C4	1.4143 (18)	C10—H10C	0.9800
C5—C6	1.518 (2)		
			
C1—N1—C2	109.23 (11)	C5—C6—H6A	109.5
C1—N2—C4	117.17 (11)	C5—C6—H6B	109.5
C4—N3—C5	122.83 (12)	H6A—C6—H6B	109.5
C4—N3—H3	119.4 (11)	C5—C6—H6C	109.5
C5—N3—H3	117.6 (11)	H6A—C6—H6C	109.5
C7—N4—C3	103.48 (10)	H6B—C6—H6C	109.5
C7—N5—C2	105.47 (10)	N4—C7—N5	114.31 (12)
C7—N5—C8	126.60 (11)	N4—C7—H7	122.8
C2—N5—C8	127.93 (11)	N5—C7—H7	122.8
N1—C1—N2	132.00 (12)	N5—C8—C10	110.62 (11)
N1—C1—Cl1	113.92 (9)	N5—C8—C9	110.24 (11)
N2—C1—Cl1	114.06 (9)	C10—C8—C9	112.42 (12)
N1—C2—N5	127.02 (11)	N5—C8—H8	107.8
N1—C2—C3	126.96 (12)	C10—C8—H8	107.8
N5—C2—C3	106.02 (11)	C9—C8—H8	107.8
C2—C3—N4	110.72 (11)	C8—C9—H9A	109.5
C2—C3—C4	116.66 (12)	C8—C9—H9B	109.5
N4—C3—C4	132.59 (12)	H9A—C9—H9B	109.5
N3—C4—N2	118.86 (12)	C8—C9—H9C	109.5
N3—C4—C3	123.20 (12)	H9A—C9—H9C	109.5
N2—C4—C3	117.94 (11)	H9B—C9—H9C	109.5
N3—C5—C6	112.64 (11)	C8—C10—H10A	109.5
N3—C5—H5A	109.1	C8—C10—H10B	109.5
C6—C5—H5A	109.1	H10A—C10—H10B	109.5
N3—C5—H5B	109.1	C8—C10—H10C	109.5
C6—C5—H5B	109.1	H10A—C10—H10C	109.5
H5A—C5—H5B	107.8	H10B—C10—H10C	109.5
			
C2—N1—C1—N2	2.0 (2)	C5—N3—C4—N2	−1.24 (19)
C2—N1—C1—Cl1	−179.87 (9)	C5—N3—C4—C3	178.29 (12)
C4—N2—C1—N1	−1.4 (2)	C1—N2—C4—N3	179.07 (11)
C4—N2—C1—Cl1	−179.56 (9)	C1—N2—C4—C3	−0.48 (17)
C1—N1—C2—N5	179.90 (12)	C2—C3—C4—N3	−178.20 (12)
C1—N1—C2—C3	−0.80 (18)	N4—C3—C4—N3	−0.3 (2)
C7—N5—C2—N1	179.21 (12)	C2—C3—C4—N2	1.33 (17)
C8—N5—C2—N1	−0.8 (2)	N4—C3—C4—N2	179.24 (13)
C7—N5—C2—C3	−0.21 (14)	C4—N3—C5—C6	−85.04 (16)
C8—N5—C2—C3	179.74 (12)	C3—N4—C7—N5	0.27 (15)
N1—C2—C3—N4	−179.04 (12)	C2—N5—C7—N4	−0.05 (15)
N5—C2—C3—N4	0.38 (14)	C8—N5—C7—N4	−179.99 (12)
N1—C2—C3—C4	−0.68 (19)	C7—N5—C8—C10	−114.50 (14)
N5—C2—C3—C4	178.74 (11)	C2—N5—C8—C10	65.56 (16)
C7—N4—C3—C2	−0.40 (14)	C7—N5—C8—C9	120.53 (14)
C7—N4—C3—C4	−178.41 (14)	C2—N5—C8—C9	−59.40 (17)

### Hydrogen-bond geometry (Å, º)

*Cg*1 is the center of gravity of the pyridine ring (C1, N1, C2, C3, C4, N2).

**Table:**

*D*—H···*A*	*D*—H	H···*A*	*D*···*A*	*D*—H···*A*
N3—H3···N4^i^	0.837 (16)	2.205 (17)	2.9979 (16)	158.2 (15)
C8—H8···*Cg*1^ii^	1.00 (1)	2.90 (1)	3.6403 (13)	131 (1)

Symmetry codes: (i) −*x*, −*y*+1, −*z*+1; (ii) −*x*+1/2, *y*+1/2, −*z*+1/2.
